# Different Neural Processes Accompany Self-Recognition in Photographs Across the Lifespan: An ERP Study Using Dizygotic Twins

**DOI:** 10.1371/journal.pone.0072586

**Published:** 2013-09-19

**Authors:** David L. Butler, Jason B. Mattingley, Ross Cunnington, Thomas Suddendorf

**Affiliations:** 1 School of Psychology, McElwain Building, University of Queensland, Saint Lucia, Australia; 2 Queensland Brain Institute, QBI Building, University of Queensland, Saint Lucia, Australia; Royal Holloway, University of London, United Kingdom

## Abstract

Our appearance changes over time, yet we can recognize ourselves in photographs from across the lifespan. Researchers have extensively studied self-recognition in photographs and have proposed that specific neural correlates are involved, but few studies have examined self-recognition using images from different periods of life. Here we compared ERP responses to photographs of participants when they were 5–15, 16–25, and 26–45 years old. We found marked differences between the responses to photographs from these time periods in terms of the neural markers generally assumed to reflect (i) the configural processing of faces (i.e., the N170), (ii) the matching of the currently perceived face to a representation already stored in memory (i.e., the P250), and (iii) the retrieval of information about the person being recognized (i.e., the N400). There was no uniform neural signature of visual self-recognition. To test whether there was anything specific to self-recognition in these brain responses, we also asked participants to identify photographs of their dizygotic twins taken from the same time periods. Critically, this allowed us to minimize the confounding effects of exposure, for it is likely that participants have been similarly exposed to each other's faces over the lifespan. The same pattern of neural response emerged with only one exception: the neural marker reflecting the retrieval of mnemonic information (N400) differed across the lifespan for self but not for twin. These results, as well as our novel approach using twins and photographs from across the lifespan, have wide-ranging consequences for the study of self-recognition and the nature of our personal identity through time.

## Introduction

The ability to recognize one's own physical appearance has generated considerable interest amongst researchers for nearly 200 years e.g., [1], [2], [3], [4], [5], [6]. Most of this attention has been driven by the assumption that this ability indicates a self-concept or self-awareness [3], [5], [7], [8] though leaner alternative interpretations have been offered [9], [10], [11]. An increasing number of researchers have searched for the neural processes associated with visual self-recognition [5]. Typically this involves comparing neural responses produced by seeing photographs of one's own face and those of others (for reviews see [12], [13], [14]). Some results have indicated that seeing these faces produces similar neural processes [15], but most report differences e.g., [16], [17], [18], [19], [20], [21], [22]. It remains contentious *how precisely* these processes actually differ [12], [13], [14]. For example, some results suggest that visual self-recognition predominantly involves the right side of the brain [17], [18], [19], [23], [24], [25], yet there are also results suggesting the left [16], [21] or both [15], [22], [26], [27], [28] sides of the brain. Furthermore, contradictory results have been reported in studies investigating the *timing* of neural processes. For instance, both similarities and differences have been reported in relation to the N170, which is a neural marker for the configural processing of facial features [29], [30], [31], [32], [33], [34], [35]. In summary, at present it is unclear whether visual self-recognition involves neural processes that are different from those involved in recognizing others, and if so, how they actually differ.

Many conflicting results, whether they involve the location or timing of neural processes, are likely due to methodological differences. Some studies used unaltered photographs of faces e.g., [15], [18] whilst others used morphs i.e., blended images involving self and others; e.g., [19], [21]. Experimental tasks have also differed, with some involving simple observation of images and others requiring identification [36], [37], [38]. Nevertheless major differences between results have emerged even when the same type of images and experimental task were used e.g., [16], [26].

### The Role of Exposure in Face Recognition

We propose that a major factor causing conflicting results between studies is exposure; that is, the duration of time that a person has seen a stimulus. Faces are processed differently according to whether they have been subject to high or low levels of exposure [39], [40], [41]. For example, it has been established that we process faces from our own race differently to those of other races depending upon the amount of exposure we have received to each race i.e., the own-race bias; [42]. Similarly, we tend to process faces of our own age differently to those of other ages (i.e., the own-age bias), and this is again likely due to the amount of exposure we have had to faces of varying ages [43], [44]. When attempting to identify the neural processes of visual self-recognition, it is therefore important to acknowledge that most people have had daily exposure to their own faces *throughout most of their lives*. For example, the effect of such exposure is shown in participants' preference for mirror-reversed (as opposed to unreversed) photographs of their own faces, and this is because most have seen their face more in mirrors than in photographs [16], [45]. Subsequently, these effects of exposure make it crucial that studies of visual self-recognition involve a control image that has been seen by participants to a similar extent as one's own image throughout the lifespan. Several attempts have been made to do so. For instance, self has been compared with similarly aged personal acquaintances, yet none come close to a lifetime of exposure [16], [35], [37], [38], [41], [46], [47], [48], [49], [50], [51].

An alternative approach has been to use participant's parents as control images [30], [41], [52]. Unfortunately, the findings from these results are likely to be confounded due to the own-age bias. Siblings have also been used, yet these comparisons may be confounded by age differences because in most of these studies the age of the siblings have not been stated [15], [20], [31], [37]. Here we overcome these issues by recruiting same aged participant pairs who are likely to have shared a similar amount of lifetime exposure to each other's faces: non-identical twins (we did not include identical twins because we wanted to reduce instances of misidentification that may have occurred due to their similarities in appearance). Such a stringent control for exposure allows us to test whether there are any unique neural processes underlying visual self-recognition, and if so, what the nature of these processes might be. We used event related potentials (ERPs) to compare participant's neural responses to photographs of their own and their twin's faces. ERPs measure brain activity at the scalp in the form of electrical amplitude as a function of time. Because millisecond resolution is attained, they afford the best opportunity to measure the neural processes for the various stages involved in face recognition [39], [40], [53]. There are four such specific stages measured by ERPs. An initial featural encoding stage occurs when facial features such as the eyes, nose, and mouth are first detected (reflected by a positive peak of amplitude at around 100 ms; i.e. the P100 [54], [55]). This is followed by a stage at which the configural relationship between these features is analyzed (reflected by a negative going peak at around 170 ms; i.e. the N170 [30], [54]). A subsequent matching stage occurs when this newly constructed representation is compared to stored structural representations (reflected by a positive peak in amplitude at around 250 ms; i.e. the P250 [32]). Finally, mnemonic information about the person is retrieved (reflected by a negative trough in amplitude between around 300–800 ms; i.e., the N400 [31], [56]). If recognizing images of self and others involves different neural processes, then we expect differences to emerge in one or more of these ERP components.

### Visual Self-recognition Across the Lifespan

Much attention has been directed at whether the neural processes associated with visual self-recognition are somehow unique, but it has rarely been considered whether the time period that the images have originated from somehow influences these neural processes (e.g., photographs taken from last year, 5 years ago, 15 years ago, etc.). Prior studies have typically only measured neural activity in response to self-images originating from one period in time (e.g., the day of the experiment). There are a number of problems with this approach. First, it does not accord with our common experience of self-recognition in photographs: we don't just see ourselves from one moment in time, but rather, from different periods of time across the lifespan (e.g., last week, when we were teenagers or children, etc). Second, if researchers want to use visual self-recognition as a means of investigating the neural processes associated with the self-concept, then it is important to ensure that participants do recognize images of self across time periods. Third, developmental and clinical evidence suggests that time period influences the neural processes of visual self-recognition. For example, children who recognize themselves in live videos may not do so in videos shown after a three-minute delay [57], [58]. There are also instances of clinical patients who, despite not being able to recognize themselves in a mirror, maintain the capacity for self-recognition in photographs from when they were younger [53], [59], [60]. Finally, an 83-year old Alzheimer's patient was only able to recognize herself in photographs when aged between 20 to 40 years [61].

**Figure 1:**
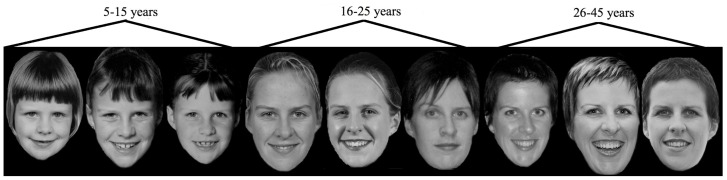
Example of images for a single person taken from different time periods. (Please note that this person has given written informed consent, as outlined in the PLOS consent form, to publication of their photograph.)

To date we note only one prior study has investigated the neural processes associated with visual self-recognition across the lifespan: different neural networks were reportedly activated for morphed images of the ‘current’ self as opposed to morphed images of the ‘childhood’ self [46]. Unfortunately such findings are questionable due to the absence of an appropriate control image involving a similar amount of exposure across the lifespan. Here we offer the first direct investigation of the neural processes associated with self-recognition across different time periods after controlling for lifetime exposure. To do so we had our dizygotic participants recognize photographs of themselves and their twin taken from across different periods of the lifespan (see Figure 1). If different neural processes are associated with visual self-recognition from different time periods, we expect to see differences emerge in one or more of the ERP components reflecting the various stages of face recognition.

## Methods and Materials

### Ethics Statement

Ethical clearance was granted from the Australian Twin Registry (2010-004) and the University of Queensland's Ethics Committee (08-PSYCH-PhD-42-CVH), both of which are in accordance with the regulations stipulated by the Australian National Health and Medical Research Council.

### Participants

Twenty people participated (two males), ranging from 26–41 years (*M* = 31.94 years, *SE* = 1.20). These were recruited in conjunction with the Australian Twin Registry (http://www.twins.org.au). All were of Caucasian descent, had normal to corrected vision, and were predominantly right handed. Participation was compensated with a $30.00 (AUS) voucher or cash.

### Stimuli and Materials

There was a total of 27 photographs involving self, twin, and an unfamiliar other matched for age and gender. Each of these identities had a total of nine photographs consisting of three each from 5–15, 16–25, and 26–45 year age periods. Photographs were obtained digitally (jpegs or bitmaps) or by scanner (for hard copies). Uniform modification involved each photograph being: (1) mirror-reversed (for self only); (2) cropped at the chin, ears, and mid-hairline; (3) adjusted in hue and luminance; (4) mounted onto a black background; (5) resized using a scale based upon a height of 100 mm; (6) converted into BMP format. To do this we used Corell Paint Shop Pro (Corell Corporation, 2003). The inter-trial stimulus consisted of a grey and white checkerboard matching the size of the area containing the faces.

The experimental task was designed and presented using E-prime software (www.pstnet.com/eprime). All instructions and images were displayed on a black background in the centre of a NEC AccuSync monitor, with a resolution of 1024×768 pixels. The viewing distance of visual stimuli from the participant was c. 90 cms. Participant responses were recorded using a standard numerical keypad (e.g., arrow up = self; arrow right = twin; arrow left = unfamiliar); these responses were counterbalanced across subjects. Response output was recorded by E-prime (for accuracy and reaction times) and Bio Semi (for EEG; http://www.bio-semi.com/).

### Experimental Task

Faces from all identities and across all time periods were presented in a pseudo-random order. To do this we divided the experiment up into six pseudo-random blocks (the order of which were counterbalanced across participants), each of which consisted of a total of 135 trials (made up from five presentations of each of the 27 images). Each face was shown for a maximum of 2000 ms, followed by the 1500 ms inter-trial stimulus. Using their right hand, participants were to respond via button press as quickly and accurately as possible to the identity of these faces. A response prior to 2000 ms would immediately result in the re-appearance of the inter-trial stimulus before going onto the next face. The total number of trials for the whole experiment was 810; this can be further broken down into 90 trials for each age period within each identity.

### Procedure

Participants were initially approached by phone via the Australian Twin Registry (ATR). If willing to participate the ATR then forwarded them a package detailing basic information about the experiment. The principal author then contacted interested people over the phone to arrange for a time to collect photographs and conduct the study. During the experiment participants were tested individually in a one-hour session in a dark room whilst sitting in a comfortable armchair. After application of the electrode cap, they were verbally and visually instructed that the experiment consisted of a series of faces being shown on the monitor; they were to respond as quickly and accurately as possible to the identity of these faces. At the beginning of each block participants were re-presented with these instructions. Before the experiment started participants engaged in a practice session involving two shortened blocks (i.e., one presentation of each of the 27 images used in the experiment).

### Electrophysiological Recording and Analyses

Electroencephalogram (EEG) data was continuously obtained using the Bio Semi ActiveTwo system (http://www.bio-semi.com/) and analysed offline using BESA software (http://www.besa.de/index_home.htm). EEG was recorded using 64 Ag-AgCl electrodes fixed within an electrode cap according to the widening International 10–20 system [62]. The use of the Bio Semi Ag-AgCl active system reduces the need for skin preparation (see http://www.bio-semi.com/). To track eye movements we recorded the vertical and horizontal electro-occulograms by placing one pair of Ag-AgCl surface electrodes supra and suborbitally to the right eye, and another pair 1 cm external to the outer canthus of each eye. EEG and electro-occulogram signals were originally sampled at 1024 Hz with a band pass filter between 0.01–100 Hz. These signals were originally referenced to the CMS and DRL electrodes during data acquisition before being re-referenced offline to the average of the 64 channels. Data were then segmented into 1250 ms epochs, with the 250 ms prior to stimulus onset being the baseline. After blink artefact correction [63], EEG data were manually searched for electro-occulogram artefacts. BESA's artefact tool was then used for rejecting trials exceeding 100 µV. Incorrect trials were further excluded from analyses. EEG waveforms were then sorted with respect to condition and averaged to create ERPs for each participant. A minimal acceptance rate of 74% of trials per condition was adopted, with most participants providing between 85% and 97% trials for each condition. ERPs were filtered with a high-pass filter of 0.1 Hz and a low-pass filter of 45 Hz (both with a slope of 12 dB/octave and of type zero phase). Grand average waveforms, averaged across all participants, were then calculated.

### Selection of Epochs and Channels for ERPs

Inspection of the grand average waveforms and topographical maps indicated, relative to baseline, the presence of the following sequence of clearly abrupt peak components over posterior regions: a positive-going peak (P100: 70–160 ms), a negative-going peak (N170: 130–245 ms), and a second positive-going peak (P250: 180–415 ms). These components were to be analysed using *peak* amplitude analyses (based upon the largest measures of amplitude from each participant within the defined epoch) as there was little ambiguity evident between participants for when the amplitude for each component typically reached their largest points. Beyond these peaks was a long negative-going trough, which whilst not abrupt, was clearly sustained (N400: 400–600 ms). This component was to be analysed using *mean* amplitude analyses (based upon the mean amount of amplitude generated from each participant within the defined epoch), for there was some ambiguity evident between participants in relation to when the amplitudes of the component reached the largest point.

Channels were selected for each component where the amplitude was maximal. Over posterior regions the channels used for each component were as follows: P100 (P7, P9, PO7, and O1 were averaged to form a proxy for the left hemisphere; a proxy for the midline was Oz; P8, P10, PO8, and O2 were averaged as a proxy for the right hemisphere); N170, P250, and N400 (left hemisphere averaged over P7, P9, and PO7; right hemisphere averaged over P8, P10, and PO8). These epochs, channels, and (hemispheric) regions are comparable to ones reported in prior self-recognition studies [29], [31], [34], [35], [51], [64].

### Statistical Analyses

All analyses were performed using repeated measures multifactorial ANOVA in SPSS (Version 17.0). Comparisons involving amplitude were coded relative to identity (self vs twin vs unfamiliar)×time period (5–15 vs 16–25 vs 26–45 years)×hemisphere (left vs midline vs right for the P100; left vs right for the N170, P250, and N400). Latencies were coded for identity×time period only in relation to the P100, N170, and P250, as all of these components involved clear, abrupt peaks. In addition we also made comparisons involving accuracy (calculated as the percentage of correct responses as a proportion of the total amount of correct and incorrect responses) and reaction time (calculated as the amount of time - in milliseconds - between the presentation of the face and the participant's response to it), both of which were coded relative to identity and time period.

Data were checked for normality using the Shapiro-Wilk test. Significant *p* values were adjusted using the Greenhouse-Geisser method for violations of sphericity, while the Bonferroni method was used for follow-up comparisons. An *a priori* approach was used for making follow up comparisons in the event of significant main effects or interactions involving time period (i.e., only self versus twin was compared). This was done because of the need to (1) ensure that self is controlled with another image subjected to similar amounts of exposure (see ‘The Role of Exposure in Face Recognition’); and (2) reduce the likelihood of reporting false negatives between self and twin by minimizing the Bonferroni correction value. See File S1 for a complete report of all statistical findings.

## Results and Discussion

**Figure 2:**
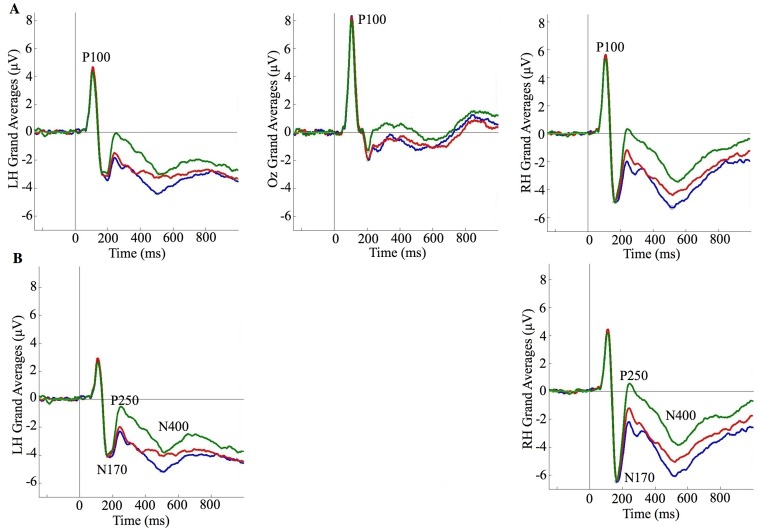
Grand average ERPs for identity. (A) P100 (LH = Left Hemisphere, Oz = Midline, RH = Right Hemisphere; see Methods and Materials for details about which channels were selected) (self = blue, twin = red, unfamiliar = green); (B) N170, P250, and N400.

Data are presented for the four ERP components proposed to reflect different stages of face processing. The grand averages and amplitudes for all conditions are presented in Figures 2 to [Fig pone-0072586-g003] [Fig pone-0072586-g004] 5.

**Figure 3 pone-0072586-g003:**
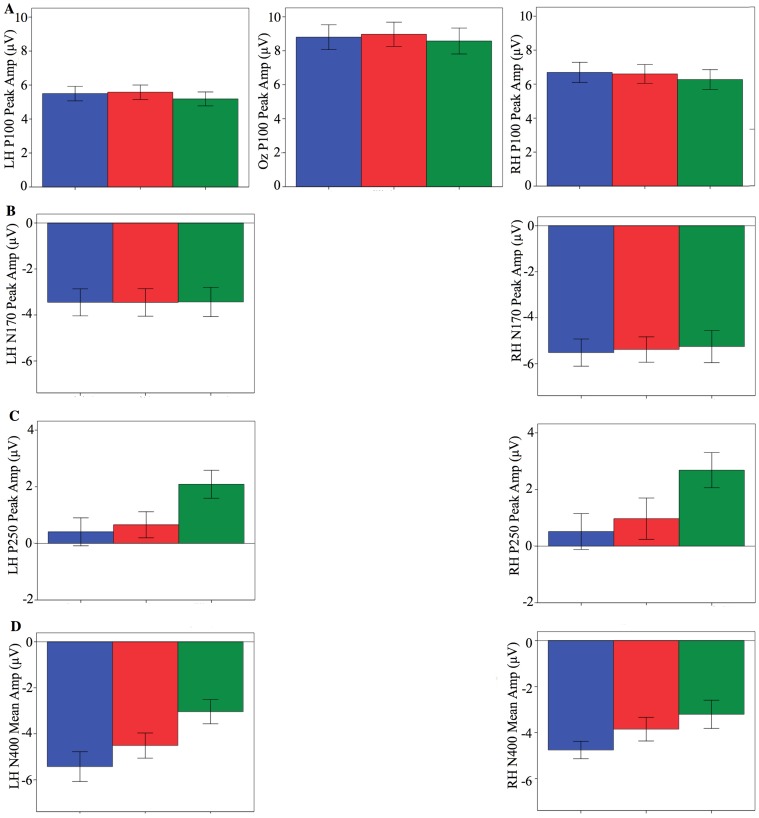
ERP bar graphs (mean and standard error) for identity. (A) P100); (B) N170; (C) P250; (D) N400. Differences emerged for the N170, P250, and N400 when comparing photographs of self and twin to an unfamiliar other. For comparisons involving self and twin there was only a difference at the N400.

**Figure 4 pone-0072586-g004:**
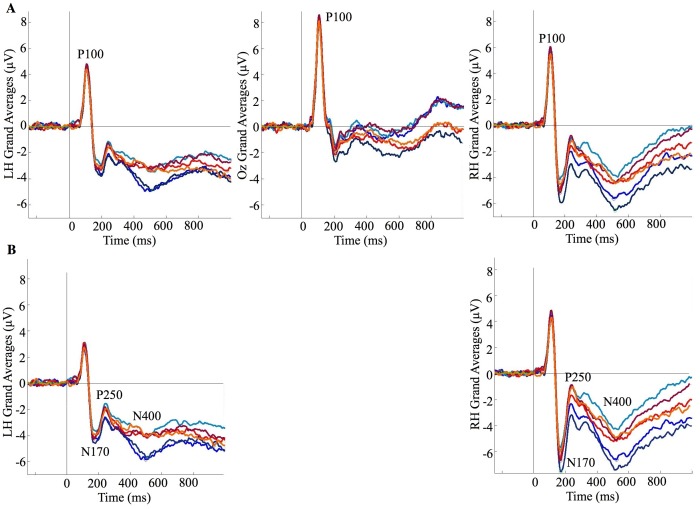
Grand average ERPs for identity×age (self 5–15 = light blue, self 16–25 = mid blue, self 26–45 = dark blue, twin 5–15 = orange, twin 16–25 = red, self 26–45 = maroon). (A) P100; (B) N170, P250, and N400.

**Figure 5:**
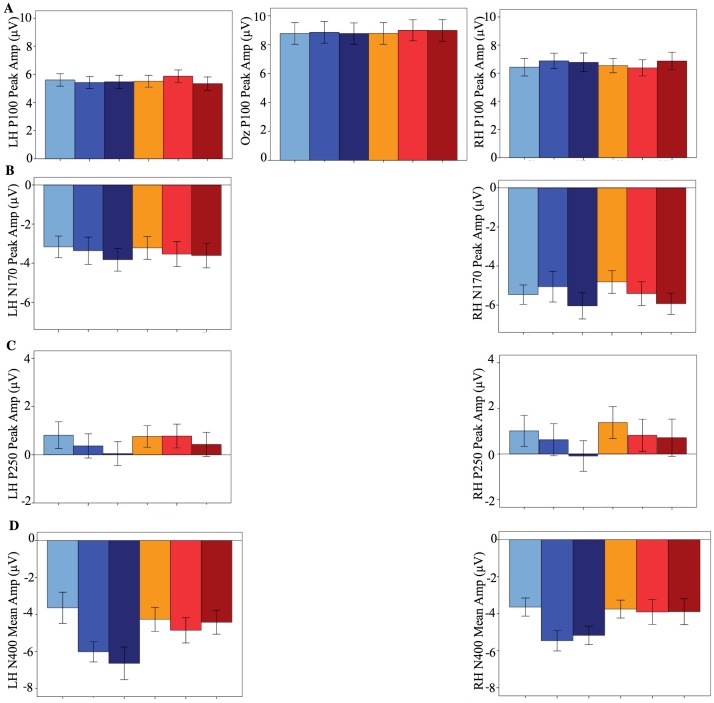
ERP bar graphs (mean and standard error) for identity×age. (A) P100; (B) N170; (C) P250; (D) N400. Differences between time periods emerged for the N170 and P250 for images of both self and twin. For the N400 differences emerged across time periods for images of self but not for twin.

We first considered the effects of the identity of the person in the photograph regardless of time period (that is, we investigated possible main effects involving identity by collapsing the data for each identity across all three time periods; see Figures 2 and 3). Compared with unfamiliar faces, both self and twin faces produced (i) a P100 with a larger peak amplitude and a longer latency, (ii) an N170 with a similar peak amplitude but longer latency, (iii) a P250 with a smaller peak amplitude but similar latency, and (iv) an N400 with a larger mean amplitude. These findings indicate that there are shared neural differences between both self *and* twin when compared to an unfamiliar other for all stages of face processing (i.e., featural, configural, matching, and mnemonic). These results are consistent with several findings that self and/or familiar faces produce differences compared to unfamiliar faces for the N170, P250, and N400 [29], [30], [31], [32], [34]. However, our findings are one of the few claiming that familiarity can influence the P100 [65], [66]. Other studies have reported that the P100 is *not* influenced by familiarity [29], [31], [34]. One reason for this apparent discrepancy - which may ultimately account for why these latter studies have failed to find familiarity effects - is the absence of data from channels typically used in P100 analyses (i.e., O1 and O2 e.g., [54], [55]; for discussion on the source of this component see Susac and colleagues [67]). Prior researchers have also noted that the P100 has been largely ignored or not reported as it was once believed to have no specific relevance to faces [54], [55]. Unfortunately, this concurs with the current ERP research involving visual self-recognition i.e.,[30], [32], [33], [35], [51]. To resolve this issue it is crucial that researchers do report the P100 with analyses based upon the appropriate channels. In any event, our results generally indicate that self and twin faces, which are associated with similar levels of high exposure, and unfamiliar faces, which are associated with low levels of exposure, involve neural differences for all stages of face recognition. Consistent with other exposure related phenomena (i.e., the own-race bias [42], the own-age bias [43], [44], and the mere exposure effect [45]), our current findings suggest that differences involving the recognition of familiar and unfamiliar faces are not being driven by identity *per se*, but rather, the amount of exposure people have had to certain faces - including their own.

When comparing self and twin faces, no differences emerged for any ERP component with the exception of the N400, which had a larger amplitude for self than twin. Therefore, self and twin faces share very similar featural, configural, and matching processes, but differ with respect to mnemonic retrieval. Consistent with prior self-recognition studies in relation to the P100 [29], [34], [68], our results reinforce the notion that self – when compared to a personally familiar other - does not involve unique featural encoding. As for the N170, previous studies have been divided about whether differences between self and familiar others do or do not exist [29], [32], [34], [35], [69]. We note that in all instances where differences have been reported there has been a failure to control for either lifetime exposure and/or age. This suggests that the lack of differences for the N170 in the present study is likely due to our control of these two related factors. For the P250, our results are contrary to most prior reports [29], [32], [34]; that is, whilst most studies have found differences between self and others, we found no such difference between self and twin. Again, our control for exposure and age is the most likely reason for this discrepancy. This is supported by one report where a substantial amount of exposure to an unfamiliar face led to similar matching processes when compared to images of self [35]. Finally, our N400 findings are generally consistent with prior results in that self is usually different from familiar others [31], [34]. Unlike the other ERP components reported here, results for the N400 suggest that identity *per se*, rather than exposure, does influence an aspect of how face recognition occurs (see below for more discussion involving potential mnemonic processes). In summary, when compared to another face associated with high levels of lifetime exposure, the neural processes of visual self-recognition are only unique in relation to mnemonic retrieval.

We next considered the effects of time period for self and twin (see Materials and Methods for discussion on these *a priori* comparisons; see Figures 4 and 5). We did this by comparing images in which participants were aged between 5–15, 16–25, and 26–45 years. No differences were observed between time periods for the P100, suggesting that the featural encoding of self and twin faces remained similar irrespective of time period. For the N170 we only observed a difference involving amplitude, however it is uncertain where this difference occurred as no comparison survived Bonferroni correction. Our tentative interpretation is that 26–45 year old images produced more peak amplitude than both other time periods (as both these comparisons approach significance), but no difference occurred between 5–15 and 16–25 year periods (as this comparison is far from significant; see File S1). These results suggest that configural processing of self and twin faces appear to be influenced by time period [70], [71]. However, whether increased N170 amplitude specifically reflects either increased *efficiency* or *difficulty* in configural processing remains contentious and requires further investigation [for discussion see 71]. As for the P250, again only differences in amplitude were observed: 5–15 year old images produced more amplitude than those in other time periods, whilst no difference was evident between 16–25 and 26–45 year old periods. Time period therefore influences the process of matching self and twin faces to their stored representations. Finally, we found that N400 amplitude differed as a function of time period, but only for images of self and not for twin. For self, 5–15 year old images produced less amplitude compared to both other time periods, whilst there was no difference between 16–25 and 26–45 year old periods. Visual self-recognition therefore appears to involve mnemonic processes that are influenced by time period. In summary, the neural processes of visual self-recognition do not remain uniform but are influenced by time period. This is consistent with developmental and clinical research where time period influences whether visual self-recognition does or does not occur [53], [57], [58], [59], [60], [61]. It is also consistent with a prior report stating that different brain regions were found when recognizing morphed images of ‘current’ self compared to ‘childhood’ self [46].

One likely explanation for the effects of time period on these neural markers associated with face recognition is the changing physical appearances that accompany aging (e.g., skin elasticity, nose and ear lengths, eye and lip shapes, etc.). Whilst not influencing featural encoding *per se*, perhaps such physical changes alter the configuration between facial features, and how the constructed representation is matched to stored representations. It is unlikely, however, that ageing cues are responsible for the effect of time period on the neural processes for mnemonic retrieval; self and twin are likely to share similar ageing cues and yet only self produced mnemonic differences between time periods. An alternative reason for the effects of time period is the amount of exposure to faces of different ages (i.e., the own-age bias) [43], [44], [70]. Again, this accounts for the differences between time periods for the configural and matching stages, but it fails to account for the difference between self and twin for mnemonic processing. Perhaps dissociated memory systems are involved as a function of identity [61], [72]. For example, seeing images of self may involve autobiographical memory, whilst seeing others involves general (i.e., semantic) memory. If so this would support the suggestion that self-recognition requires a self-concept [3], [5], [7], [8]. Future research should address how it is that time period influences self-recognition and its neural processes.

Comparing self and twin also allowed us to consider one of the proposed mechanisms underlying kin recognition; namely, self-referent phenotype matching (i.e., we recognize our kin by implicitly or explicitly comparing the similarity of other people's appearance to our own [73]). Two predictions follow from this proposal: people will show (1) more prosocial behaviour towards images that resemble themselves; (2) less sexual attraction to those who resemble themselves. Evidence is accruing in support of both predictions [74], [75], [76]. However, there is a more fundamental prediction stemming from this ‘self-referent phenotype’ theory: if we recognize kin by matching their appearance to our own, then we should expect that as the degree of similarity between self and kin increases the degree of similarity in the neural processes for recognizing self and kin should also increase [37]. Our current results involving non-identical twins suggests this may be so, for compared to an unfamiliar (and unrelated) person, participants showed similar neural processes for the featural encoding, configural, and matching stages when recognizing images of self and their twin. However, confirmation of this prediction ultimately requires a more direct test comparing participants who view non-identical twins with those who view identical (i.e., monozygotic) twins. We would expect that compared to non-identical participants, identical participants should show more similarity in their neural processes for recognizing themselves and their twin.

In conclusion, we demonstrated that when the amount of lifetime exposure to self and other faces is similar, visual self-recognition generally only involves unique neural processes in relation to mnemonic retrieval. We also demonstrated that time period influences the neural processes of self-recognition. Future investigations can further address the effects of time period on the neural processes of self-recognition by using our lifespan paradigm to confirm whether differences emerge in the location of brain areas [46]. It would also be worth investigating whether animals that recognize themselves in mirrors are also able to recognize themselves across time in photographs or videos. Answering such questions should further our knowledge about visual self-recognition and its relationship, if any, to the self-concept and complex psychological processes such as self-awareness and kin-recognition.

## Supporting Information

File S1 **There is a full report of all statistical comparisons available in the online version of this paper.**
(DOCX)

## References

[1] Darwin C (1872/1998) The Expression of Emotions in Man and the Animals. Oxford: Oxford University Press.

[2] Darwin C (1877) A biographical sketch of an infant. Mind 2: 285–294.

[3] Gallup G (1970) Chimpanzees: Self recognition. Science 167: 86–87. 4982211 10.1126/science.167.3914.86

[4] Grant J (1828) Account of the structure, manners, and habits of an orang-utan from Borneo, in possession of George Swinton. Edinburgh Journal of Science 9: 1–24.

[5] Keenan J, Gallup G, Falk D (2004) The Face in the Mirror: How We Know Who We Are. New York: Harper Perennial.

[6] Preyer W (1889) The Mind of the Child Part II: The Development of the Intellect. New York, NY: Appleton.

[7] Gallup G (1982) Self awareness and the emergence of mind in primates. American Journal of Primatology 2: 237–248. 10.1002/ajp.1350020302 32192237

[8] Gallup G (1985) Do minds exist in species other than our own? Neuroscience and Biobehavioral Reviews 9: 631–641. 4080281 10.1016/0149-7634(85)90010-7

[9] Mitchell R (1997) A comparison of the self awareness and kinaesthetic-visual matching theories of self recognition: Autistic children and others. In: Snodgrass J, Thompson R, editors. The Self Across Psychology: Self Recognition, Self Awareness, and the Self Concept. New York: New York Academy of Sciences. pp. 39–62. 10.1111/j.1749-6632.1997.tb48245.x 9237464

[10] Nielsen M, Suddendorf T, Slaughter V (2006) Mirror self-recognition beyond the face. Child Development 77: 176–185. 16460532 10.1111/j.1467-8624.2006.00863.x

[11] Suddendorf T, Whiten A (2001) Mental evolution and development: evidence for secondary representation in children, great ages, and other animals. Psychological Bulletin 127: 629–650. 11548971 10.1037/0033-2909.127.5.629

[12] Devue C, Bredart S (2011) The neural correlates of visual self recognition. Consciousness and Cognition 20: 40–51. 20880722 10.1016/j.concog.2010.09.007

[13] Gillihan S, Farah M (2005) Is self special? A critical review of evidence from experimental psychology and cognitive neuroscience. Psychological Bulletin 131: 76–97. 15631554 10.1037/0033-2909.131.1.76

[14] Platek S, Wathne K, Tierney N, Thomson J (2008) Neural correlates of self-face recognition: An effect location meta-analysis. Brain Research 1232: 173–184. 18656465 10.1016/j.brainres.2008.07.010

[15] Sperry R, Zaidel E, Zaidel D (1979) Self recognition and social awareness in the deconnected minor hemisphere. Neuropsychologia 17: 153–166. 379688 10.1016/0028-3932(79)90006-x

[16] Brady N, Campbell M, Flaherty M (2004) My left brain and me: A dissociation in the perception of self and others. Neuropsychologia 42: 1156–1161. 15178167 10.1016/j.neuropsychologia.2004.02.007

[17] Keenan J, Ganis G, Freund S, Pascual-Leone A (2000) Hand response differences in a self-face identification task. Neuropsychologia 38: 1047–1053. 10775715 10.1016/s0028-3932(99)00145-1

[18] Keenan J, McCutcheon B, Freund S, Gallup G, Sanders G, et al (1999) Left hand advantage in a self-face recognition task. Neuropsychologia 37: 1421–1425. 10606015 10.1016/s0028-3932(99)00025-1

[19] Keenan J, Nelson A, O'Connor M, Pascual-Leone A (2001) Self-recognition and the right hemisphere. Nature 409: 305. 11201730 10.1038/35053167

[20] Preilowski B (1977) Self recognition as a test of consciousness in left and right hemisphere of “split-brain” patients. Activatas Nervosa Superior (Praha) 19: 343–344. 551649

[21] Turk D, Heatherton T, Kelley W, Funnell M, Gazzaniga M, et al (2002) Mike or me? Self-recognition in a split brain patient. Nature Neuroscience 5: 841–842. 12195428 10.1038/nn907

[22] Uddin L, Rayman J, Zaidel E (2005) Split brain reveals separate but equal self recognition in the two cerebral hemispheres. Consciousness and Cognition 14: 633–640. 16091274 10.1016/j.concog.2005.01.008

[23] Keenan J, Ganis G, Freund S, Pascual-Leone A (2000) Self-face identification is increased with left hand responses. Laterality 5: 259–268. 15513146 10.1080/713754382

[24] Keenan J, Wheeler M, Platek S, Lardi G, Lassonde M (2003) Self-face processing in a callosotomy patient. European Journal of Neuroscience 18: 2391–2395. 14622201 10.1046/j.1460-9568.2003.02958.x

[25] Uddin L, Molnar-Szakacs I, Zaidel E, Iacoboni M (2006) RTMS to the right inferior parietal lobule disrupts self-other discrimination. Social Cognitive and Affective Neuroscience 1: 65–71. 17387382 10.1093/scan/nsl003 PMC1832105

[26] Brady N, Campbell M, Flaherty M (2005) Perceptual asymmetries are preserved in memory for highly familiar faces of self and friend. Brain and Cognition 58: 334–342. 15963384 10.1016/j.bandc.2005.01.001

[27] Ma Y, Han S (2009) Self-face advantage is modulated by social threat - Boss effect on self-face recognition. Journal of Experimental Social Psychology 45: 1048–1051.

[28] Keyes H, Brady N (2010) Self-face recognition is characterized by “bilateral gain” and by faster, more accurate performance which persists when faces are inverted. The Quarterly Journal of Experimental Psychology 63: 840–847. 20198537 10.1080/17470211003611264

[29] Caharel S, Bernard C, Thibaut F, Haouzir S, Maggio-Clozel C, et al (2007) The effects of familiarity and emotional expression on face processing examined by ERPs in patients with schizophrenia. Schizophrenia Research 95: 186–196. 17644314 10.1016/j.schres.2007.06.015

[30] Caharel S, Courtay N, Bernard C, Lalonde R, Rebai M (2005) Familiarity and emotional expression influence an early stage of face processing: An electrophysiological study. Brain and Cognition 59: 96–100. 16019117 10.1016/j.bandc.2005.05.005

[31] Caharel S, Fiori N, Bernard C, Lalonde R, Rebai M (2006) The effects of inversion and eye displacements of familiar and unknown faces on early and late-stage ERPs. International Journal of Psychophysiology 62: 141–151. 16678927 10.1016/j.ijpsycho.2006.03.002

[32] Caharel S, Poiroux S, Bernard C, Thibaut F, Lalonde R, et al (2002) ERPs associated with familiarity and degree of familiarity during face recognition. International Journal of Neuroscience 112: 1499–1512. 12652901 10.1080/00207450290158368

[33] Gunji A, Inagaki M, Inoue E, Takeshima Y, Kaga M (2009) Event-related potentials of self-face recognition in children with pervasive developmental disorders. Brain and Development 31: 139–147. 18590948 10.1016/j.braindev.2008.04.011

[34] Keyes H, Brady N, Reilly R, Foxe J (2010) My face or yours? Event-related potential correlates of self-face processing. Brain and Cognition 72: 244–254. 19854553 10.1016/j.bandc.2009.09.006

[35] Tanaka J, Curran T, Porterfield A, Collins D (2006) Activation of preexisting and acquired face representations: The N250 event-related potential as an index of face familiarity. Journal of Cognitive Neuroscience 18: 1488–1497. 16989550 10.1162/jocn.2006.18.9.1488

[36] Platek S, Keenan J, Gallup G, Mohamed F (2004) Where am I? The neurological correlates of self and other. Cognitive Brain Research 19: 114–122. 15019708 10.1016/j.cogbrainres.2003.11.014

[37] Platek S, Kemp S (2009) Is family special to the brain? An event related fMRI study of familiar, familial, and self face recognition. Neuropsychologia 47: 849–858. 19159636 10.1016/j.neuropsychologia.2008.12.027

[38] Platek S, Loughead J, Busch S, Ruparel K, Phend N, et al (2006) Neural substrates for functionally discriminating self face from personally familiar faces. Human Brain Mapping 27: 91–98. 16035037 10.1002/hbm.20168 PMC6871291

[39] Bruce V, Young A (1986) Understanding face recognition. British Journal of Psychology 77: 305–327. 3756376 10.1111/j.2044-8295.1986.tb02199.x

[40] Gobbini M, Haxby J (2007) Neural systems for recognition of familiar faces. Neuropsychologia 45: 32–41. 16797608 10.1016/j.neuropsychologia.2006.04.015

[41] Taylor M, Arsalidou M, Bayless S, Morris D, Evans J, et al (2009) Neural correlates of personally familiar faces: parents, partner, and own faces. Human Brain Mapping 30: 2008–2020. 18726910 10.1002/hbm.20646 PMC6870744

[42] Meissner C, Brigham J (2001) Thirty years of investigating the own-race bias in memory for faces - a meta-analytic review. Psychology Public Policy and Law 7: 3–35.

[43] Harrison V, Hole G (2009) Evidence for a contact-based explanation of the own-age bias in face recognition. Psychonomic Bulletin and Review 16: 264–269. 19293092 10.3758/PBR.16.2.264

[44] Hills P, Lewis M (2011) The own-age face recognition bias in children and adults. The Quarterly Journal of Experimental Psychology 64: 17–23. 21213196 10.1080/17470218.2010.537926

[45] Mita T, Dermer M, Knight J (1977) Reversed facial images and the mere-exposure hypothesis. Journal of Personality and Social Psychology 35: 597–601.

[46] Apps M, Tajadura-Jimenez A, Turley G, Tsakiris M (2012) The different faces of one's self: An fMRI study into the recognition of current and past self-facial appearances. Neuroimage 63: 1720–1729. 22940117 10.1016/j.neuroimage.2012.08.053 PMC3772343

[47] Kircher T, Senior C, Phillips M, Benson P, Bullmore E, et al (2000) Towards a functional neuroanatomy of self processing: Effects of faces and words. Cognitive Brain Research 10: 113–144. 10.1016/s0926-6410(00)00036-7 10978701

[48] Kircher T, Senior C, Phillips M, Rabe-Hesketh S, Benson P, et al (2001) Recognizing one's own face. Cognition 78: B1–B15. 11062324 10.1016/s0010-0277(00)00104-9

[49] Sugiura M, Watanabe J, Maeda Y, Matsue Y, Fukuda H, et al (2005) Cortical mechanisms of visual self-recognition. Neuro Image 24: 143–149. 15588605 10.1016/j.neuroimage.2004.07.063

[50] Sugiura M, Sassa Y, Jeong H, Miura N, Akitsuki Y, et al (2006) Multiple brain networks for visual self-recognition with different sensitivity for motion and body part. Neuro Image 32: 1905–1917. 16806977 10.1016/j.neuroimage.2006.05.026

[51] Sui J, Zhu Y, Han S (2006) Self-face recognition in attended and unattended conditions: An event-related brain potential study. Neuroreport 17: 423–427. 16514370 10.1097/01.wnr.0000203357.65190.61

[52] Greenberg S, Goshen-Gottstein Y (2009) Not all faces are processed equally: Evidence for featural rather than holistic processing of one's own face in a face-imaging task. Journal of Experimental Psychology: Learning, Memory, and Cognition 35: 499–508. 10.1037/a0014640 19271862

[53] Breen N, Caine D, Coltheart M (2001) Mirrored-self misidentification: Two cases of focal onset dementia. Neurocase 7: 239–254. 11459919 10.1093/neucas/7.3.239

[54] Itier R, Taylor M (2002) Inversion and contrast polarity reversal affect both encoding and recognition processes of unfamiliar faces: A repetition study using ERPs. Neuroimage 15: 353–372. 11798271 10.1006/nimg.2001.0982

[55] Herrmann M, Ehlis A, Ellgring H, Fallgatter A (2005) Early stages (P100) of face perception in humans as measured with event-related potentials (ERPs). Journal of Neural Transmission 112: 1073–1081. 15583954 10.1007/s00702-004-0250-8

[56] Bentin S, Deouell L (2000) Structural encoding and identification in face processing: ERP evidence for separate mechanisms. Cognitive Neuropsychology 17: 35–54. 20945170 10.1080/026432900380472

[57] Povinelli D, Landau K, Perilloux H (1996) Self-recognition in young children using delayed versus live feedback: evidence for a developmental asynchrony. Child Development 67: 1540–1554. 8890499

[58] Suddendorf T (1999) Children's understanding of the relation between delayed video representation and current reality: A test for self-awareness? Journal of Experimental Child Psychology 72: 157–176. 10047437 10.1006/jecp.1998.2485

[59] Phillips M, Howard R, David A (1996) “Mirror, mirror on the wall, who…?”: Towards a model of visual self recognition. Cognitive Neuropsychiatry 1: 153–164. 16571480 10.1080/135468096396613

[60] Villarejo A, Martin V, Moreno-Ramos T, Camacho-Salas A, Porta-Etessam J, et al (2010) Mirrored-self misidentifcation in a patient without dementia: Evidence for a right hemispheric and bifrontal damage. Neurocase 17: 276–284. 10.1080/13554794.2010.498427 20812138

[61] Hehman J, German T, Klein S (2005) Impaired self-recognition from recent photographs in a case of late-stage alzheimer's disease. Social Cognition 23: 118–123.

[62] American-Clinical-Neurophysiological-Society (2006) Guideline 5: Guidelines for standard electrode position nomenclature. Journal of Clinical Neurophysiology 23: 107–110. 16612226 10.1097/00004691-200604000-00006

[63] Semlitsch H, Anderer P, Schuster P, Presslich O (1986) A solution for reliable and valid reduction of ocular artifacts. Psychophysiology 23: 695–703. 3823345 10.1111/j.1469-8986.1986.tb00696.x

[64] Rossion B, Jacques C (2008) Does physical interstimulus variance account for early electrophysiological face sensitive responses in the human brain? Ten lessons on the N170. Neuro Image 39. 10.1016/j.neuroimage.2007.10.011 18055223

[65] Seeck M, Michel C, Mainwaring N, Cosgrove R, Blume H, et al (1997) Evidence for rapid recognition from human scalp and intracranial electrodes. Neuroreport 8: 2749–2754. 9295112 10.1097/00001756-199708180-00021

[66] Bruno Debruille J, Guillem F, Renault B (1998) ERPs and chronometry of face recognition: Following Seeck et al. and George et al. Neuroreport 9: 3349–3353. 9855278 10.1097/00001756-199810260-00002

[67] Susac A, Ilmoniemi R, Pihko E, Nurminen J, Supek S (2009) Early dissociation of face and object processing: A magnetoencephalographic study. Human Brain Mapping 30: 917–927. 18344191 10.1002/hbm.20557 PMC6870775

[68] Sui J, Liu C, Han S (2009) Cultural difference in neural mechanisms of self-recognition. Social Neuroscience 4: 402–411. 19739032 10.1080/17470910802674825 PMC3348608

[69] Tanaka J, Porterfield A (2001) The search for self-identity: The own-face effect (Abstract). Journal of Vision 1: 334.

[70] Melinder A, Gredeback G, Westerlund A, Nelson C (2010) Brain activation during upright and inverted encoding of own- and other-age faces: ERP evidence for an own-age bias. Developmental Science 13: 588–598. 20590723 10.1111/j.1467-7687.2009.00910.x PMC2898522

[71] Weise H, Schweinberger S, Hansen K (2008) The age of the beholder: ERP evidence of an age-own bias in face memory. Neuropsychologia 46: 2973–2985. 18602408 10.1016/j.neuropsychologia.2008.06.007

[72] Klein S, Gangi C (2010) The multiplicity of self: Neuropsychological evidence and its implications for the self as a construct in psychological research. Annals of the New York Academy of Sciences 1191: 1–15. 20392272 10.1111/j.1749-6632.2010.05441.x

[73] Daly M (1982) Whom are new born babies said to resemble? Ethology and Sociobiology 3: 69–78.

[74] DeBruine L (2002) Facial resemblance enhances trust. Proceedings of the Royal Society of London B: Biological Sciences 271: 1307–1312. 10.1098/rspb.2002.2034 PMC1691034 12079651

[75] DeBruine L (2004) Resemblance to self increases the appeal of child faces to both men and women. Evolution and Human Behavior 25: 142–154.

[76] DeBruine L (2005) Trustworthy but not lust-worthy: Context-specific effects of facial resemblance. Proceedings of the Royal Society of London B: Biological Sciences 272: 919–922. 10.1098/rspb.2004.3003 PMC1564091 16024346

