# Supplementary material for: Different Neural Processes Accompany Self-Recognition in Photographs Across the Lifespan: An ERP Study Using Dizygotic Twins
Source: PLoS One. 2013 Sep 19;8(9):e72586. doi: 10.1371/journal.pone.0072586 (PMC3777976; doi:10.1371/journal.pone.0072586)
Supplement: File S1 — There is a full report of all statistical comparisons available in the online version of this paper. (DOCX) [file pone.0072586.s001.docx]

SI for “Different Neural Processes Accompany Self-recognition in Photographs Across the Lifespan: An Event Related Potential Study Using Dizygotic Twins”

**Authors:** David L. Butler, Jason B. Mattingley, Ross Cunnington, and Thomas Suddendorf

**Results**

Accuracy of responses was influenced by identity (*F*(1.512, 28.723) = 4.430, *p*. = .019, *η*^2^ = .189). Participants were less accurate when recognizing photographs of twin (*M* = 97.37%, *SE* = .58) compared to an unfamiliar other (*M* = 98.80%, *SE* = .30; *t*(19) = -2.686, *p*. = .015). No differences occurred between self (*M* = 98.30%, *SE* = .32) and twin (*t*(19) = 1.629, *p*. = .120), or self and unfamiliar (*t*(19) = -1.549, *p*. = .138). Time period also influenced accuracy (*F*(2, 38) = 4.432, *p*. = .019, *η*^2^ = .189). Less accurate responses occurred for 5-15 year old photographs (*M* = 97.54%, *SE* = .46) compared to 26-45 year old photographs (*M* = 98.52%, *SE* = .34; *t*(19) = -2.713, *p*. = .014). No differences occurred between 5-15 and 16-25 year old photographs (*M* = 98.41%, *SE* = .32; *t*(19) = -2.027, *p*. = .057) or 16-25 and 26-45 year old photographs (*t*(19) = -.403, *p*. = .691). There was an interaction between identity and time period (*F*(2.425, 46.084) = 3.554, *p*. = .029, *η*^2^ = .158). For self, 5-15 year old photographs (*M* = 97.00%, *SE* = .79) were recognized less accurately than 26-45 year old photographs (*M* = 99.22%, *SE* = .27; *t*(19) = -3.162, *p*. = .005). No other differences emerged within identity across time periods (self 5-15 vs self 16-25 (*M* = 98.67%, *SE* = .29): *t*(19) = -1.949, *p*. = .066; self 16-25 vs self 26-45: *t*(19) = -1.422, *p*. = .171; twin 5-15 (*M* = 97.00%, *SE* = .60) vs twin 16-25 (*M* = 97.28%, *SE* = .72): *t*(19) = -.567, *p*. = .577; twin 5-15 vs twin 26-45 (*M* = 97.83%, *SE* = .63): *t*(19) = -1.510, *p*. = .148; twin 16-25 vs twin 26-45: *t*(19) = -1.097, *p*. = .287; unfamiliar 5-15 (*M* = 98.61%, *SE* = .46) vs unfamiliar 16-25 (*M* = 99.28%, *SE* = .23): *t*(19) = -1.831, *p*. = .083; unfamiliar 5-15 vs unfamiliar 26-45 (*M* = 98.50%, *SE* = .37): *t*(19) = .252, *p*. = .804; unfamiliar 16-25 vs unfamiliar 26-45: *t*(19) = 3.036, *p*. = .007).

For speed participants were no faster when responding to photographs of self (*M* = 687.74 ms, *SE* =21.79), twin (*M* = 700.24 ms, *SE* = 18.57), and an unfamiliar other (*M* = 673.40 ms, *SE* =16.85; *F*(2, 38) = 2.945, *p*. = .065; *η*^2^ = .134). There was a main effect for time period (*F*(1.103, 20.951) = *p.* = .001; *η*^2^ = .445). Twenty-six to 45 year old images (*M* = 670.39 ms, *SE* = 16.61) were recognized faster than 16-25 year old images (*M* = 684.38 ms, *SE* = 17.83; *t*(19) = 5.910, *p*. = .000) and 5-15 year old images (*M* = 706.60, *SE* = 20.76; *t*(19) = 4.276, *p*. = .000), with 16-25 and 5-15 year old images also differing (*t*(19) = 3.017, *p*. = .007). There was no interaction between identity and time period (*F*(2.791, 53.03) = 1.790, *p*. = .164; *η*^2^ = .086).

**P100**

There was a main effect for identity (*F*(2, 38) = 11.941, *p*. = .000; *η*^2^ = .386). Both self (*M* = 7.00 μV, *SE* = .52; *t*(19) = 3.730, *p.* = .001) and twin (*M* = 7.05 μV, *SE* = .50; *t*(19) = 5.631, *p*. = .000) produced a larger P100 when compared to an unfamiliar other (*M* = 6.68 μV, *SE* = .52). There was no difference between self and twin (*t*(19) = -.556, *p*. = .585). No main effect was found for time period (*F*(2, 38) = .509, *p*. = .605; *η*^2^ = .026). A main effect was found for hemisphere (*F*(2, 38) = 24.117, *p.* = .000; *η*^2^ = .559). The midline (Oz; *M* = 8.78 μV, *SE* = .73) showed a significantly greater amplitude than both the right (*M* = 6.52 μV, *SE* = .57; *t*(19) = -4.627, *p*. = .000) and left hemispheres (*M* = 5.42 μV, *SE* = .42; *t*(19) = -6.362, *p*. = .000), with no significant difference being found between the right and left hemispheres (*t*(19) = 2.388, *p*. = .027).

There were no interactions between either identity and time period (*F*(4, 76) = .128, *p.* = .972; *η*^2^ = .007), time period and hemisphere (*F*(4, 76) = 1.213, *p*. = .312; *η*^2^ = .060), identity and hemisphere (*F*(4, 76) = .586, *p*. = .673; *η*^2^ = .030), or between identity and time period and hemisphere (*F*(8, 152) = 1.912, *p.* = .062).

For P100 latency no main effects were found for identity (*F*(2, 38) = 2.436, *p*. = .101; *η*^2^ = .114) or time period (*F*(2, 38) = .942, *p*. = .399; *η*^2^ = .047). An interaction occurred between identity and time period (*F*(4, 76) = 2.676, *p*. = .038; *η*^2^ = .123). There were no differences across time periods for self (self 5-15: *M* = 112.07 ms, *SE* = 1.49; self 16-25: *M* = 110.10 ms, *SE* = 2.06; *t*(19) = 1.399, *p*. = .178; self 5-15 vs 26-45: *M* = 111.65 ms, *SE* = 1.46; *t*(19) = .357, *p*. = .725; self 16-25 vs 26-45: *t*(19) = -.778, *p*. = .446) or twin (twin 5-15: *M* = 111.17 ms, *SE* = 1.86; twin 16-25: *M* = 110.60 ms, *SE* = 1.80; *t*(19) = .550, *p*. = 589; twin 5-15 vs 26-45: *M* = 111.62 ms, *SE* = 1.53; *t*(19) = -.364, *p*. = .720; twin 16-25 vs 26-45: *t*(19) = -.853, *p*. = 404). To find the source of the interaction comparisons across time periods were also made for unfamiliar; no differences were found (unfamiliar 5-15: *M* = 107.10 ms, *SE* = 1.75; unfamiliar 16-25: *M* = 110.92, *SE* = 1.46; *t*(19) = -2.246, *p*. = .037; unfamiliar 5-15 vs 26-45: *M* = 110.70 ms, *SE* = 1.53; *t*(19) = -2.353, *p*. = .030; unfamiliar 16-25 vs 26-45: *t*(19) = .195, *p*. = .847). The source of the interaction is most likely to involve comparisons across identity, and because this is not consistent with our emphasis on the effects of time period within identity, these comparisons are not included here.

**N170**

Violation of normality assumptions lead to a square-root transformation of the data for N170 amplitude. No main effect was found for identity (*F*(2, 38) = .218, *p.* = .805; *η*^2^ = .011). A main effect was found for time period (*F*(2, 38) = 3.512, *p.* = .040; *η*^2^ = .156), yet follow-up comparisons using Bonferroni adjustments revealed non-significant differences between five to 15 (*M* = -5.69 μV, *SE* = .72) versus 16-25 (*M* = -5.63 μV, *SE* = .69; *t*(19) = 0.326, *p*. = .748), 16-25 versus 26-45 (*M* = -5.99 μV, *SE* = .65; *t*(19) = -2.074, *p*. = .052), and five to 15 versus 26-45 year old photographs (*t*(19) = -2.208, *p*. = .040). A main effect was found for hemisphere (*F*(1, 19) = 14.557, *p.* = .001; *η*^2^ = .434), with the right (*M* = -7.50 μV, *SE* = .99) showing a larger N170 than the left (*M* = -4.04. μV, *SE* = .63).

No interaction was observed between identity and time period (*F*(1.786, 33.927) = 1.254, *p*. = .295; *η*^2^ = .062), identity and hemisphere (*F*(2, 38) = .293, *p.* = .748; *η*^2^ = .015), time period and hemisphere (*F*(2, 38) = .343, *p.* = .712; *η*^2^ = .018), or identity and time period and hemisphere (*F*(2.837, 53.985) = 1.323, *p.* = .277; *η*^2^ = .065).

For N170 latency (which did not involve transformed data) a main effect was found for identity (*F*(2, 38) =7.708, *p.* = .002; *η*^2^ = .289). Both self (*M* = 176.78 ms, *SE* = 3.80; *t(*19) = 3.447, *p.* = .003) and twin (*M* = 175.27 ms, *SE* = 3.69; *t(*19) = 2.837, *p.* = .011) produced later N170s compared to unfamiliar (*M* = 171.30 ms, *SE* = 3.46). No difference between self and twin was observed (*t*(19) = 1.136, *p*. = .270). No main effect was found for time period (*F*(2, 38) = 1.540, *p*. = .227; *η*^2^ = .075). No interaction was found between identity and time period (*F*(4, 76) = .622, *p.* = .648; *η*^2^ = .032).

**P250**

A main effect occurred for identity (*F*(2, 38) = 48.111, *p.* = .000; *η*^2^ = .717). Both self (*M* = .46 μV, *SE* = .48; *t(*19) = -7.505, *p.* = .000) and twin (*M* = .81 μV, *SE* = .53; *t(*19) = -8.589, *p.* = .000) produced a smaller P250 compared with an unfamiliar other (*M* = 2.38 μV, *SE* = .51). No difference was found between self and twin (*t(*19) = -1.974, *p.* = .063). A main effect was also found for time period (*F*(2, 38) = 10.869, *p.* = .000; *η*^2^ = .364). Five to 15 year old photographs (*M* = 1.54 μV, *SE* = .54) produced a larger amplitude compared with both 16-25 (*M* = 1.13 μV, *SE* = .47 *t(*19) = 3.188, *p.* = .005) and 26-45 year old photographs (*M* = .99 μV, *SE* = .49; *t(*19) = 5.496, *p.* = .000). No difference occurred between 16-25 and 26-45 year old photographs (*t(*19) = 1.052, *p.* = .306). No main effect was found for hemisphere (*F*(1, 19) = .401, *p.* = .534; *η*^2^ = .021).

No interactions were observed between identity and time period (*F* (2.620, 49.778) = 1.475, *p.* = .218; *η*^2^ = .072), identity and hemisphere (*F*(2, 38) = .1.332, *p.* = .276; *η*^2^ = .066), time period and hemisphere (*F*(2, 38) = 1.392, *p.* = .261; *η*^2^ = .068), or identity and time period and hemisphere (*F*(4, 76) = 1.414, *p.* = .238; *η*^2^ = .069).

For P250 latency no main effect was found for identity (*F*(2, 38) = 1.073, *p.* = .352; *η*^2^ = .053). No difference in time period was observed (*F*(2, 38) = .518, *p.* = .600; *η*^2^ = .027). An interaction was observed between identity and time period (*F*(2.683, 50.968) = 3.421, *p.* = .028; *η*^2^ = .153; self 5-15: *M* = 283.96 ms, *SE* = 10.86; self 16-25: *M* = 283.23 ms, *SE* = 11.33; self 26-45: *M* = 283.87 ms, *SE* = 11.94; twin 5-15: *M* = 288.11 ms, *SE* = 11.43; twin 16-25: *M* = 283.38 ms, *SE* = 10.32; twin 26-45: *M* = 262.82 ms, *SE* = 9.66; unfamiliar 5-15: *M* = 275.79 ms, *SE* = 8.99; unfamiliar 16-25: *M* = 290.01 ms, *SE* = 9.94; unfamiliar 26-45: *M* = 294.58 ms, *SE* = 8.99). Within self and twin images, there were no differences between any time periods (self 5-15 vs 16-25: *t*(19) = .075, *p*. = .941; self 5-15 vs 26-45: *t*(19) = .013, *p*. = .990; self 16-25 vs 26-45: *t*(19) = -.052, *p*. = .959; twin 5-15 vs 16-25: *t*(19) = .603, *p*. = .554; twin 5-15 vs 26-45: *t*(19) = 2.276, *p*. = .035; twin 16-25 vs 26-45: *t*(19) = 2.436, *p*. = .025). To find the location of the interaction further comparisons were performed. Within unfamiliar photographs, there was a difference between 5-15 and 16-25 year old periods only (*t*(19) = -3.473, *p*. = .003; unfamiliar 5-15 vs 26-45: *t*(19) = -2.509, *p*. = .021; unfamiliar 16-25 vs 26-45: *t*(19) = -.645, *p*. = .526).

**N400**

A main effect was found for identity (*F*(2, 38) = 17.602, *p*. = .000; *η*^2^ = .481). Self (*M* = -5.10 μV, *SE* = .38; *t*(19) = -5.676, *p.* = .000) and twin (*M* = - 4.18 μV, *SE* = .40; *t*(19) = -3.185, *p.* = .000) both produced larger N400s compared with an unfamiliar other (*M* = -3.13. μV, *SE* = .44). Self also produced a significantly larger N400 compared with twin (*t*(19) = -2.876, *p*. = . 010). A main effect was found for time period (*F*(2, 38) = 8.468, *p.* = .001; *η*^2^ = .308). Five to 15 year old photographs (*M* = -3.42 μV, *SE* = .38) produced a significantly smaller N400 compared with both 16-25 (*M* = -4.46 μV, *SE* = .39; *t*(19) = 3.439, *p*. = .003) and 26-45 year old photographs (*M* = - -4.53 μV, *SE* = .41; *t*(19) = 4.514, *p*. = .000). There was no difference between 16-25 and 26-45 year old photographs (*t*(19) = .205, *p*. = .839). There was no main effect for hemisphere (*F*(1, 19) = 0.317, *p*. = .580; *η*^2^ = .016).

An interaction was observed between identity and time period (*F*(4, 76) = 2.497 *p.* = .050; *η*^2^ = .116; self 5-15: *M* = -3.64 μV, *SE* = .52; self 16-25: *M* = -5.74 μV, *SE* = .38; self 26-45: *M* = -5.91 μV, *SE* = .57; twin 5-15: *M* = -4.01 μV, *SE* = .45; twin 16-25: *M* = -4.38 μV, *SE* = .53; twin 26-45: *M* = -4.16 μV, *SE* = .51; unfamiliar 5-15: *M* = -2.61, μV, *SE* = .51; unfamiliar 16-25: *M* = -3.25, μV, *SE* = .47; unfamiliar 26-45: *M* = -3.52, μV, *SE* = .58). Within self images, 5-15 year old photographs produced a smaller amplitude compared with both 16-25 (*t*(19) = 3.907, *p*. = .001) and 26-45 year old photographs (*t*(19) = 4.517, *p.* = .000), with no difference between 16-25 and 26-45 year old photographs (*t*(19) = .261, *p.* = .797). Within twin there were no differences between 5-15 (*M* = -4.01 μV, *SE* = .45) and 16-25 year old photographs (*M* = -4.38 μV, *SE* = .53; *t*(19) = .849, *p.* = .407), 5-15 and 26-45 year old photographs (*M* = -4.16 μV, *SE* = .51; *t*(19) = .287, *p*. = .777), nor 16-25 and 26-45 year old photographs (*t*(19) = -.397, *p.* = .696). No interaction occurred between identity and hemisphere (*F*(2, 38) = 2.499, *p.* = .096; *η*^2^ = .116), nor between time period and hemisphere (*F*(2, 38) = 1.728, *p.* = .191; *η*^2^ = .083), or identity and age and hemisphere (*F*(4, 76) = 0.670, *p.* = .615; *η*^2^ = .034).
